# Stability of health-related quality of life and morbidity burden from 18 months after diagnosis of prostate cancer: results of a UK-wide population-based outcome cohort

**DOI:** 10.1007/s00520-021-06650-7

**Published:** 2021-12-13

**Authors:** Samantha J. Mason, Amy Downing, Sarah Wilding, Luke Hounsome, Penny Wright, Eila Watson, Richard Wagland, Hugh Butcher, Paul Kind, Peter Selby, Anna Gavin, Adam W. Glaser

**Affiliations:** 1 School of Human and Health Sciences, University of Huddersfield, Huddersfield, UK; 2 Leeds Institute of Medical Research at St James’s, University of Leeds, Leeds, UK; 3 Leeds Institute for Data Analytics, University of Leeds, Level 11, Worsley Building, Leeds, LS2 9NL UK; 4 School of Psychology, University of Leeds, Leeds, UK; 5 Public Health England, Bristol, UK; 6 Oxford Institute of Nursing, Midwifery and Allied Health Research, Oxford Brookes University, Oxford, UK; 7 Faculty of Health Sciences, University of Southampton, Southampton, UK; 8 Academic Unit of Health Economics, University of Leeds, Leeds, UK; 9 Leeds Teaching Hospitals NHS Trust, Leeds, UK; 10 Northern Ireland Cancer Registry, Queens University Belfast, Belfast, UK

**Keywords:** Prostate cancer, Patient-reported outcomes, Survivorship, Health-related quality of life, Health status, Functional outcomes

## Abstract

**Objective:**

To evaluate the dynamic nature of self-reported health-related quality of life (HRQL) and morbidity burden in men diagnosed with prostate cancer, we performed a follow-up study of the Life After Prostate Cancer Diagnosis (LAPCD) study cohort 12 months after initial survey.

**Methods:**

The LAPCD study collected information from 35,823 men across the UK who were 18–42 months post-diagnosis of prostate cancer. Men who were still alive 12 months later were resurveyed. Generic HRQL (EQ-5D-5L plus self-assessed health rating) and prostate cancer-specific outcomes (EPIC-26) were assessed. Treatment(s) received was self-reported. Previously defined clinically meaningful differences were used to evaluate changes in outcomes over time.

**Results:**

A total of 28,450 men across all disease stages completed follow-up surveys (85.8% response). Of the 21,700 included in this study, 89.7% reported no additional treatments since the first survey. This group experienced stable urinary and bowel outcomes, with good function for most men at both time points. On-going poor (but stable) urinary issues were associated with previous surgery. Sexual function scores remained low (mean: 26.8/100). Self-assessed health ratings were stable over time. The largest declines in HRQL and functional outcomes were experienced by men reporting their first active treatment between surveys.

**Discussion:**

The results suggest stability of HRQL and most specific morbidities by 18–42 months for men who report no further treatment in the subsequent 12 months. This is reassuring for those with good function and HRQL but re-enforces the need for early intervention and support for men who experience poor outcomes.

**Supplementary Information:**

The online version contains supplementary material available at 10.1007/s00520-021-06650-7.

## Introduction

Men are living for increasing periods with and beyond a diagnosis of prostate cancer (PCa) [1]. In light of this, focus has shifted to understanding the needs of men surviving PCa and their health-related quality of life (HRQL) in the years following diagnosis and treatment [2].

It is believed that the most severe treatment side effects occur in the first year following treatment for PCa, with some improvement thereafter [3–6]. Studies have found that surgery has the greatest detrimental impact on urinary continence and erectile function, radiotherapy is most associated with bowel and urinary irritation problems [3–6] and androgen deprivation therapy (ADT) has a range of adverse side effects, such as sexual dysfunction, fatigue and problems with emotional wellbeing [7–9].

Many HRQL studies focus on the impact of specific primary treatments, typically in men with localised PCa [3, 4, 7, 10]. Few studies have addressed longer-term outcomes [7, 11], particularly in patients treated with a range of regimens and those with advanced disease.

The UK-wide Life After Prostate Cancer Diagnosis (LAPCD) study [12] adopted an established approach to the measurement of population-level HRQL, previously used in a national population of colorectal cancer survivors [13]. Over 35,000 men 18–42 months post-diagnosis completed the first LAPCD survey, and results showed that while HRQL was generally good, a high level of sexual dysfunction was experienced across the cohort and substantial problems with hormonal function and fatigue were reported, particularly by men treated with ADT [14]. Results supported previous findings showing that surgical patients experienced the worst continence and radiotherapy patients reported more bowel issues than other treatment groups [14].

Given that the most severe side effects of PCa treatment are reported during the first year, it might be assumed that the acute consequences of initial treatment, particularly surgery and radiation, will have settled to a stable level by 18 months post-diagnosis. However, little is known about whether HRQL remains stable, improves or deteriorates in the medium to long-term. To evaluate the dynamic nature of self-reported HRQL and morbidity burden, we performed a follow-up study of the LAPCD cohort approximately 12 months after completion of the initial survey.

## Patients and methods

### Sample

The LAPCD study design has been detailed previously [12]. Briefly, men alive 18–42 months after a PCa diagnosis were invited to participate in the first LAPCD survey from October 2015 to November 2016. They were identified through national cancer registration systems in England, Wales and Northern Ireland. Patients from Scotland were identified through hospital activity data. Men were sent postal surveys on behalf of their NHS provider and consented by returning completed surveys. Men who completed the first survey were re-surveyed 12 months later. Up to two reminders were sent to non-responders. Before survey mail-outs and reminders, a death check was carried out to ensure that men who had recently died were not contacted. The study received ethics and governance approvals from the following organisations: Newcastle and North Tyneside 1 Research Ethics Committee (15/NE/0036), Confidentiality Advisory Group (15/CAG/0110), NHS Scotland Public Benefit and Privacy Panel (0516–0364) and NHS Research and Development approval from Wales, Scotland and Northern Ireland.

### Survey content

Survey content was the same at both time points, except for questions about how men were diagnosed, employment status at diagnosis and ethnicity, which were not included in the follow-up survey as they would not have changed. Questions were included about treatments received, generic HRQL (EQ-5D-5L [15, 16]) and PCa specific outcomes (Expanded Prostate Cancer Index Composite-26 [EPIC-26 [17]]) along with sociodemographic information and presence of other long-term conditions (LTCs).

EQ-5D-5L records problems on five dimensions (mobility, self-care, usual activities, pain/discomfort and anxiety/depression), plus a rating of self-assessed health (SAH) based on ‘how good or bad your health is today’ (valued 0–100, where 100 represents best possible health). There are five response options for the domains ranging from no problems to extreme problems.

EPIC-26 measures function over five domains (urinary incontinence, urinary irritation and obstruction, bowel function, sexual function and vitality and hormonal function). Domain scores range from 0 to 100, with 100 representing best possible function. Items are scored on either a four or five point scale [18].

### Data analysis

Stage at diagnosis was obtained from national cancer registration records and categorised as I/II (localised), III (locally advanced) and IV (metastatic). An area-based measure of socio-economic deprivation (split into quintiles) was derived using postcode of residence [19–22]. Age (at first survey) and treatment were derived from the survey data. Age was grouped into < 55 years, 55–64 years, 65–74 years, 75–85 years and ≥ 85 years.

**Fig. 1:**
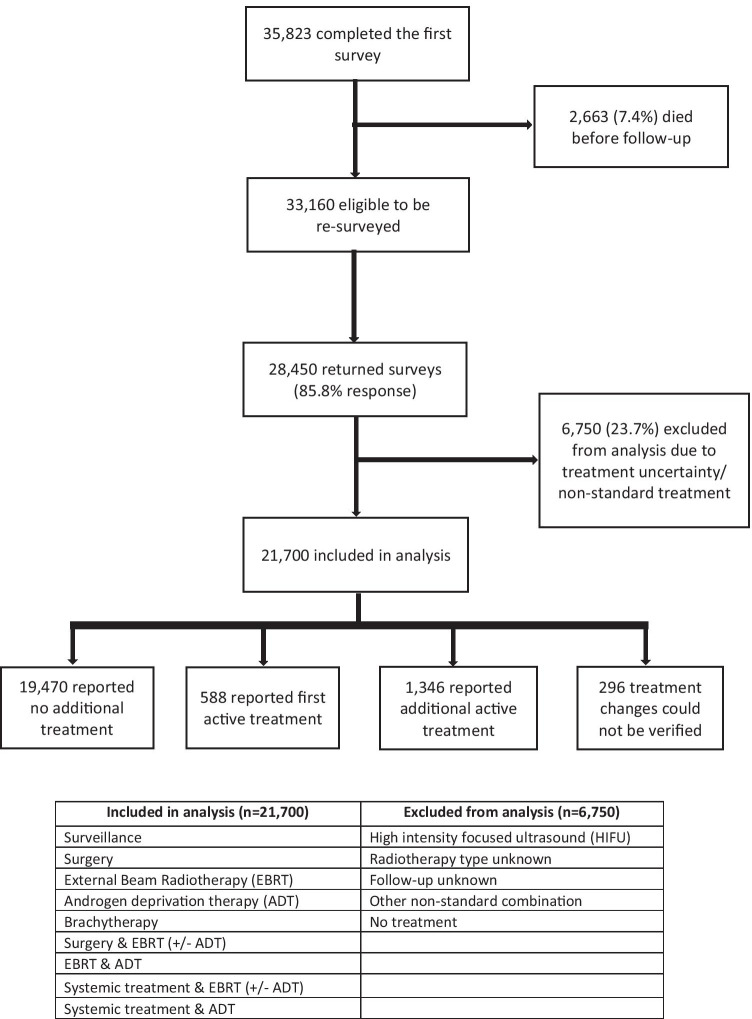
Flow diagram of inclusions and exclusions

Information on treatment(s) was taken from the questionnaire and grouped into single therapies (e.g. surgery or external beam radiotherapy [EBRT]) or combination therapies (e.g. EBRT and ADT). Analysis was restricted to men who reported receiving one of the most common single or combination treatments, as reported in earlier LAPCD work [14] (Fig. 1)and excluded those who were unsure about what treatment they received or reported a non-standard combination of treatments. Respondents were categorised into three groups: those who self-reported no further treatment at the time of the follow-up survey, those who reported receiving their first active treatment at follow-up (and were previously on active surveillance (AS) or watchful waiting (WW)) and those who reported receiving additional active treatment at follow-up.

For EQ-5D-5L, the proportion of respondents reporting any problem, regardless of severity, in each dimension separately and across all five dimensions was derived. Mean SAH ratings were calculated. To compare changes in SAH scores between the two surveys, a previously defined clinically meaningful difference (CMD) of 7 points was used [23].

Mean scores were calculated for each EPIC-26 domain. In addition, individual item responses were used to derive the proportion of respondents that reported a moderate/big problem (or equivalent) [24]. To compare changes in EPIC-26 domain scores over time, previously defined figures representing CMDs were used [25]. In addition, men who reported poor functional outcomes at first survey (EPIC-26 domain scores of < 50, apart from the sexual domain where scores < 10 were used) were analysed separately. These scores were below the average domain scores and thus represented poor function.

Descriptive statistics were used to report respondent characteristics, EQ-5D-5L and EPIC-26 responses. Outcomes were analysed in relation to age, stage and type of treatment. Analyses were based on patients who provided answers to questions in both the original and follow-up surveys unless otherwise stated. Analyses were performed using Stata version 15 (Stata, College Station, TX).

## Results

### Sample size and response rates

Of the 35,823 men who completed the initial survey, 2663 (7.4%) men died in the period between surveys, giving a final sample of 33,160 men eligible to complete the follow-up survey. Of these, 28,450 returned completed surveys (85.8% response rate) (Fig. 1).

Men who did not complete the follow-up survey were more likely to have advanced disease (stage IV at diagnosis), be ≥ 85 years old and have reported ≥ 4 LTCs in the first survey.

### Characteristics of the study population

Analyses were performed on 21,700 men who reported receiving one or more of the single or combination therapies detailed in Fig. 1 in both surveys. Table 1 details the characteristics of these men. Half of the cohort (49.5%) were aged 65–74 at the first survey, and over half (56.9%) had stage I or II disease at diagnosis, 20.3% had stage III and 9.1% had stage IV cancer.

**Table 1:** Patient and tumour characteristics split by treatment status at follow-up

Characteristic		No additional treatment (***n*** = 19,470)		AS/WW to first active treatment (***n*** = 588)		Additional active treatment (***n*** = 1346)		***P***	Overall (***n*** = 21,404)	
		***n***	%	***n***	%	***n***	%		***N***	%
Stage at diagnosis	I/II	11,179	57.4	408	69.4	602	44.7	< 0.001	12,189	56.9
	III	3989	20.5	25	4.3	339	25.2		4353	20.3
	IV	1627	8.4	40	6.8	269	20.0		1936	9.0
	Unknown	2675	13.7	115	19.6	136	10.1		2926	13.7
Age at first survey*	< 55 years	401	2.1	7	1.2	21	1.6	< 0.001	429	2.0
	55–64 years	3377	17.3	89	15.1	162	12.0		3628	17.0
	65–74 years	9688	49.8	251	42.7	663	49.3		10,602	49.5
	75–84 years	5376	27.6	184	31.3	453	33.7		6013	28.1
	85 + years	626	3.2	57	9.7	47	3.5		730	3.4
Quintile of socio-economic deprivation	1 — least deprived	5510	28.3	149	25.3	364	27.0	0.405	6023	28.1
	2	5248	27.0	151	25.7	380	28.2		5779	27.0
	3	3987	20.5	138	23.5	281	20.9		4406	20.6
	4	2685	13.8	78	13.3	181	13.5		2944	13.8
	5 — most deprived	1598	8.2	58	9.9	115	8.5		1771	8.3
	Unknown	442	2.3	14	2.4	25	1.9		481	2.2

^*^Age was unknown for 2 men

Most men reported no additional treatments since the first survey (19,470/21,700, 89.7%), including 15.4% (3039/19,740) who reported no active treatment (AS or WW) at both time points. The remaining 10.3% (2230/21,700) reported additional treatment at follow-up. We were unable to verify the treatment changes reported by 296 respondents (Supplementary table 1). These respondents were excluded from analysis leaving 1934 men who received additional treatment (Table 1). Of these 1934 men, 588 (30.4%) reported no active treatment at first survey (AS or WW) and active treatment at follow-up: the most common subsequent treatments were surgery alone (*n* = 234), ADT alone (*n* = 172) and combined EBRT and ADT (*n* = 78). A further 29% (560/1934) of men reported the addition of ADT following initial EBRT treatment, 16.1% (312/1934) reported moving to systemic treatment and 12.9% (250/1934) reported additional EBRT and ADT following surgery (Supplementary table 2).

### Respondents who reported no additional active treatment at follow-up

#### Urinary and bowel function

Mean urinary incontinence scores were high in the original (82.8/100) and follow-up surveys (81.9/100), indicating good function. Surgical treatment had the largest impact on continence, with this group reporting the lowest scores in the original and follow-up surveys (73.9/100 and 74.2/100 respectively for the surgery alone group). No CMDs in scores were observed across age, stage, or treatment groups (Table 2). Poor continence (score < 50) was reported by 10% of men (*n* = 1683) in the first survey. At follow-up, 70% of these men continued to report poor continence (mean score 28.8) (Table 3).

**Table 2:** Functional outcomes (EPIC-26 domain scores) in survey 1 and survey 2

	Urinary incontinence				Urinary irritation				Bowel function				Hormone function				Sexual function			
	***n***	Survey 1	Survey 2	Diff	***n***	Survey 1	Survey 2	Diff	***n***	Survey 1	Survey 2	Diff	***n***	Survey 1	Survey 2	Diff	***n***	Survey 1	Survey 2	Diff
No additional treatment reported																				
Overall	16,887	82.8	81.9	− 0.9	15,058	87.3	87.5	0.2	16,303	90.1	90.1	0.0	16,945	82.1	84.2	2.1	17,386	26.5	26.8	0.3
Stage I/II	9770	82.6	81.9	− 0.7	8717	87.3	87.5	0.2	9445	90.6	90.8	0.2	9732	85.4	87.1	1.7	10,068	30.9	31.0	0.1
Stage III	3482	81.8	81.1	− 0.7	3086	87.8	88.1	0.3	3323	88.3	88.4	0.1	3472	77.2	80.4	3.2	3566	17.8	19.2	1.4
Stage IV	1351	85.2	83.5	− 1.7	1211	85.3	85.4	0.1	1308	89.7	89.3	− 0.4	1418	69.9	72.8	2.9	1395	13.1	13.6	0.5
< 55 years	380	81.5	82.4	0.9	365	87.0	87.8	0.8	382	90.6	90.9	0.3	380	82.3	84.4	2.1	391	46.6	49.7	3.1
55–64 years	3148	80.3	79.8	− 0.5	2975	87.0	87.4	0.4	3146	90.7	90.9	0.2	3161	82.4	84.3	1.9	3239	36.5	37.7	1.2
65–74 years	8605	82.2	81.5	− 0.7	7700	87.8	88.1	0.3	8328	90.4	90.7	0.3	8702	83.3	85.4	2.1	8927	26.3	26.7	0.4
75–84 years	4319	85.4	84.3	− 1.1	3660	86.6	86.6	0.0	4049	88.8	88.5	− 0.3	4287	80.0	82.1	2.1	4409	18.9	18.3	− 0.6
> 85 years	433	85.0	81.4	− 3.6	357	86.2	83.7	− 2.5	397	90.6	88.9	− 1.7	414	77.5	77.9	0.4	419	13.1	12.8	− 0.3
Monitoring	2591	88.5	86.9	− 1.6	2357	84.5	84.0	− 0.5	2577	93.9	93.6	− 0.3	2595	91.1	90.9	− 0.2	2634	47.8	45.5	− 2.3
Brachytherapy alone	713	90.9	90.6	− 0.3	665	83.8	87.5	3.7	697	89.7	90.7	1.0	711	91.2	91.6	0.4	722	47.6	46.7	− 0.9
Surgery alone	4821	73.9	74.2	0.3	4268	91.1	91.2	0.1	4623	94.5	94.5	0.0	4735	90.3	90.5	0.2	5008	26.5	27.3	0.8
Surgery and EBRT (± ADT)	1426	73.8	72.4	− 1.4	1232	87.0	86.6	− 0.4	1355	86.9	87.2	0.3	1430	78.3	80.3	2.0	1498	16.9	17.2	0.3
EBRT alone	814	86.8	86.0	− 0.8	688	87.0	87.9	0.9	765	86.6	87.1	0.5	794	85.8	88.1	2.3	872	28.1	27.1	− 1.0
EBRT and ADT	4769	87.6	86.7	− 0.9	4311	86.3	86.7	0.4	4610	84.5	84.9	0.4	4895	73.0	78.3	**5.3**	4904	19.2	21.0	1.8
ADT alone	1265	87.8	85.1	− 2.7	1096	86.1	85.2	− 0.9	1205	92.2	91.4	− 0.8	1285	70.8	72.3	1.5	1262	13.2	12.5	− 0.7
Systemic therapy and ADT	256	88.0	85.7	− 2.3	233	85.9	85.1	− 0.8	247	93.3	91.2	− 2.1	263	69.8	70.9	1.1	254	12.3	12.7	0.4
Systemic therapy and EBRT (± ADT)	232	85.5	82.9	− 2.6	208	83.8	84.4	0.6	224	85.3	84.1	− 1.2	237	68.8	72.7	3.9	232	15.2	16.7	1.5
Additional treatment reported																				
Overall	1600	84.9	80.8	− 4.1	1425	85.3	85.3	0.0	1560	89.6	87.8	− 1.8	1624	79.8	78.9	− 0.9	1644	26.4	20.3	− 6.1
***Monitoring to first active treatment***																				
Surgery*	207	87.8	75.9	** − 11.9**	185	83.0	87.1	4.1	197	93.4	93.8	0.4	199	91.4	89.5	− 1.9	209	50.5	25.6	** − 24.9**
EBRT or brachytherapy**	113	86.9	83.2	− 3.7	111	86.8	80.2	** − 6.6**	117	92.9	84.6	** − 8.3**	116	88.4	78.2	** − 10.2**	117	46.5	28.6	** − 17.9**
ADT***	237	84.7	80.1	− 4.6	209	83.4	80.9	− 2.5	225	91.2	87.5	− 3.7	232	84.4	74.7	** − 9.7**	228	32.3	19.3	** − 13.0**
***Additional active treatment reported***																				
Surgery to surgery and EBRT (± ADT)	229	74.4	71.4	− 3.0	197	91.4	88.1	− 3.3	218	93.4	86.7	** − 6.7**	221	89.1	84.1	** − 5.0**	234	24.1	22.3	− 1.8
EBRT to EBRT and ADT	465	87.1	86.6	− 0.5	404	85.8	87.6	1.8	441	86.4	87.4	1.0	473	77.8	81.7	3.9	487	23.2	23.0	− 0.2
EBRT and ADT to surgery and EBRT (± ADT)	47	83.9	77.9	** − 6.0**	45	81.7	82.6	0.9	49	82.2	82.0	− 0.2	54	67.6	71.6	**4.0**	55	12.9	12.2	− 0.7
EBRT and ADT to systemic therapy and EBRT	101	87.1	80.4	** − 6.7**	92	81.2	81.4	0.2	110	82.1	82.2	0.1	111	66.6	66.4	− 0.2	103	16.5	12.0	− 4.5
ADT to systemic therapy and ADT	106	91.0	87.3	− 3.7	99	88.3	87.4	− 0.9	111	93.3	93.0	− 0.3	122	69.8	71.3	1.5	108	12.1	11.3	− 0.8
ADT to EBRT and ADT	68	88.0	83.8	− 4.2	66	80.6	81.5	0.9	71	90.4	84.6	** − 5.8**	73	72.7	74.7	2.0	70	18.5	14.4	− 4.1

Due to the large number of men included in the study, statistical significance can be achieved with only small differences in outcomes, and these may not be clinically relevant. Results should be considered alongside previously estimated clinically meaningful differences. For EPIC-26: urinary incontinence (6–9 points); urinary irritation/obstruction (5–7 points); bowel function (4–6 points); vitality/hormone function (4–6 points); sexual function (10–12 points). Bold denotes clinically meaningful difference in score

*EBRT* external beam radiotherapy, *ADT* androgen deprivation therapy

^*^Group includes surgery alone and surgery with EBRT & ADT

^**^Group includes EBRT, EBRT and ADT and brachytherapy treatments

^***^Group includes ADT alone, EBRT an ADT, ADT and systemic and surgery and EBRT and ADT treatments

**Table 3:** Percentage of men reporting poor functional outcomes in survey 1 and survey 2 (in men who reported no further treatment at follow-up)

	Total no. men	Poor function in survey 1			Poor function in survey 2		
		*n*	%^a^	Mean	*n*	%^b^	Mean
Urinary incontinence	16,887	1683	10.0	32.1	1177	69.9	28.8
Urinary irritation	15,058	439	2.9	42.4	161	36.7	38.9
Bowel function	16,303	678	4.2	37.5	325	47.9	35.5
Hormone function	16,945	1669	9.9	37.2	821	49.2	35.3
Sexual function	17,386	5968	34.3	2.8	4243	71.1	2.3

^*^The threshold for poor function is a score < 50 for urinary incontinence, urinary irritation, bowel function and hormone function and a score of < 10 for sexual function

^a^Percentage of men who scored below the threshold* at survey 1, where the denominator is men who reported no additional treatment at follow-up

^b^Percentage of men who continued to score below the threshold* at survey 2, where the denominator is men who scored below the threshold at survey 1

Overall, bowel function scores were high with no observed change at follow-up (90.1/100 in both surveys) (Table 2). Compared to other domains, a small proportion of men reported poor bowel scores (< 50) in the first survey (4.2%, 678 men, mean score 37.5). Around half of these men (48%) reported continued poor bowel function scores (mean score 35.5) at follow-up (Table 3).

#### Vitality and hormone function

The largest improvements at follow-up were reported in this domain, with increases in scores across all stages and age groups. Men treated with combined EBRT and ADT reported a CMD in hormone function at follow-up (+ 5.3 points, mean score 78.3) (Table 2). Fewer men indicated they had moderate/big problems with hot flushes (16% at follow-up compared to 29% in the original survey) and changes in body weight (17% compared to 22% in the original survey). Low hormone domain scores (< 50) were reported by 10% of respondents (1669, mean score 37.2) in the first survey. At follow-up, 50.7% of this group continued to report low scores in this domain (mean score 35.1) (Table 3).

#### Sexual function

Mean scores for sexual function remained poor at follow-up (+ 0.3 points, mean score 26.8), with scores much lower than for other domains in both the first and follow-up surveys (Table 2). In terms of perceived ‘bother’, similar numbers reported their (lack of) sexual function to be a moderate or big problem (44.9% in the original survey and 44.1% at follow-up). One-third of men scored < 10/100 in the first survey (34.3%; mean score 2.8). Of these, 71.1% continued to report very poor sexual function at follow-up (mean score 2.3) (Table 3). Men treated with ADT reported the worst sexual function at follow-up (mean scores ranged from 12.5 for ADT alone to 21.0 for combined EBRT and ADT) (Table 2). Men treated with ADT also reported the largest proportion of ‘poor/very poor’ responses when asked about their ability to have an erection (89%).

#### Generic HRQL

There were small increases (1–3%) in the proportion of men reporting problems at follow-up on the EQ-5D-5L dimensions, except for anxiety/depression. Overall SAH was stable over time (decreasing 0.2 points to 78.9) and across age, stage and treatment groups. Respondents diagnosed with stage IV cancer and those aged ≥ 85 years reported more problems in all EQ-5D dimensions and worse SAH at follow-up, although these differences were not clinically meaningful (Table 4).

**Table 4:** Health-related quality of life (% reporting any level of problem in each EQ-5D dimension and self-assessed health rating) in survey 1 and survey 2

	Mobility				Self-care				Usual activities				Pain/Discomfort				Anxiety/Depression				% reporting ≥ 1 HRQL problem				**Mean SAH rating**			
	***n***	Survey 1 (%)	Survey 2 (%)	Diff	***n***	Survey 1 (%)	Survey 2 (%)	Diff	***n***	Survey 1 (%)	Survey 2 (%)	Diff	***n***	Survey 1 (%)	Survey 2 (%)	Diff	***n***	Survey 1 (%)	Survey 2 (%)	Diff	***n***	Survey 1 (%)	**Survey 2 (%)**	**Diff**	***n***	**Survey 1**	**Survey 2**	**Diff**
No additional treatment reported																												
Overall	19,025	29.7	32.9	3.2	19,042	9.8	12.6	2.8	18,989	32.2	34.7	2.5	18,954	37.7	39.7	2.0	18,943	30.5	30.3	− 0.2	18,518	57.5	58.5	1.0	18,869	79.1	78.9	− 0.2
Stage I/II	10,938	26.7	29.4	2.7	10,935	8.8	10.8	2.0	10,911	29.0	31.5	2.5	10,883	36.2	37.8	1.6	10,891	29.4	28.7	− 0.7	10,648	55.3	56.0	0.7	10,845	79.8	79.9	0.1
Stage III	3886	31.7	35.1	3.4	3901	10.3	13.1	2.8	3887	34.8	36.7	1.9	100	38.5	40.1	1.6	3871	31.7	32.0	0.3	3793	59.6	59.9	0.3	3,862	78.5	78.2	− 0.3
Stage IV	1588	42.3	47.6	5.3	1592	14.1	20.8	6.7	1588	45.5	48.1	2.6	1587	46.6	50.8	4.2	1579	36.6	36.7	0.1	1541	68.2	71.2	3.0	1,577	75.9	74.8	− 1.1
< 55 years	395	12.2	10.9	− 1.3	395	4.8	6.3	1.5	394	23.6	20.8	− 2.8	394	33.8	33.8	0.0	395	44.1	43.8	− 0.3	389	59.6	59.1	− 0.5	388	80.0	80.2	0.2
55–64 years	3336	20.7	21.6	0.9	3342	8.2	9.3	1.1	3335	27.7	26.9	− 0.8	3334	35.2	33.4	− 1.8	3323	37.1	35.9	− 1.2	3273	55.2	53.6	− 1.6	3,306	79.6	80.1	0.5
65–74 years	9502	25.2	27.7	2.5	9513	8.1	10.0	1.9	9484	27.9	29.7	1.8	9463	35.4	37.1	1.7	9453	28.9	28.5	− 0.4	9270	53.7	54.0	0.3	9,430	80.4	80.4	0.0
75–84 years	5193	41.0	46.5	5.5	5189	12.9	17.5	4.6	5173	40.4	45.7	5.3	5161	42.8	46.4	3.6	5169	27.9	28.0	0.1	5008	63.4	66.6	3.2	5,150	77.1	76.4	− 0.7
> 85 years	597	65.7	76.1	10.4	601	23.0	33.6	10.6	602	60.1	71.3	11.2	600	46.8	61.7	14.9	601	30.6	37.6	7.0	577	78.9	86.3	7.4	593	71.6	68.4	− 3.2
Monitoring	2962	26.8	29.3	2.5	2961	8.8	11.7	2.9	2961	25.5	30.2	4.7	2947	33.4	38.6	5.2	2958	28.8	27.7	− 1.1	2889	53.6	56.3	2.7	2,954	80.3	80.0	− 0.3
Brachytherapy alone	750	14.0	16.9	2.9	750	3.2	5.2	2.0	751	16.0	16.8	0.8	749	31.0	30.8	− 0.2	747	24.8	24.6	− 0.2	736	45.9	45.2	− 0.7	747	83.5	83.4	− 0.1
Surgery alone	5215	18.5	20.8	2.3	5219	6.3	7.2	0.9	5202	24.1	25.0	0.9	5200	30.4	30.8	0.4	5190	27.1	28.0	0.9	5107	48.5	49.0	0.5	5,157	82.1	82.2	0.1
Surgery and EBRT (± ADT)	1617	30.0	32.9	2.9	1623	10.5	14.4	3.9	1619	35.4	37.4	2.0	1611	38.9	39.3	0.4	1614	34.5	36.7	2.2	1573	60.7	61.5	0.8	1,597	78.1	77.7	− 0.4
EBRT alone	1001	32.7	36.7	4.0	998	9.5	12.1	2.6	999	30.7	35.2	4.5	991	39.7	41.5	1.8	988	23.5	24.2	0.7	965	56.3	57.6	1.3	983	79.5	79.5	0.0
EBRT and ADT	5362	35.8	38.1	2.3	5369	11.5	13.6	2.1	5345	37.8	38.9	1.1	5338	43.8	44.2	0.4	5333	33.0	31.4	− 1.6	5202	63.1	62.9	− 0.2	5,335	77.2	77.4	0.2
ADT alone	1559	52.2	60.4	8.2	1561	19.2	26.8	7.6	1556	52.1	59.5	7.4	1560	46.7	53.1	6.4	1558	35.4	35.9	0.5	1501	73.4	77.0	3.6	1,539	73.0	71.6	− 1.4
Systemic therapy and ADT	297	46.1	54.6	8.5	297	12.8	25.3	12.5	296	49.0	58.1	9.1	296	48.7	59.8	11.1	295	41.7	41.0	− 0.7	293	74.1	79.5	5.4	296	74.4	72.1	− 2.3
Systemic therapy and EBRT (± ADT)	262	42.0	52.7	10.7	264	13.6	22.7	9.1	260	48.9	52.7	3.8	262	47.7	52.7	5.0	260	39.2	35.8	− 3.4	252	72.6	72.2	− 0.4	261	74.7	73.5	− 1.2
Additional treatment reported																												
Overall	1873	33.7	40.3	6.6	1875	12.0	16.0	4.0	1872	35.2	42.8	7.6	1870	41.9	46.3	4.4	1863	34.9	36.3	1.4	1830	63.5	67.1	3.6	1,880	77.8	76.1	− 1.7
***Monitoring to first active treatment***																												
Surgery*	236	26.3	26.3	0.0	236	9.3	12.7	3.4	237	26.6	30.8	4.2	236	38.1	37.7	− 0.4	235	33.2	31.1	− 2.1	232	56.9	56.5	− 0.4	239	78.9	78.9	0.0
EBRT or brachytherapy**	134	23.9	30.6	6.7	133	6.0	9.8	3.8	135	24.4	31.9	7.5	135	32.6	48.9	16.3	133	33.1	32.3	− 0.8	130	55.4	67.7	12.3	134	79.1	77.2	− 1.9
ADT***	277	42.6	49.5	6.9	277	17.3	20.6	3.3	277	41.2	54.9	13.7	276	39.9	51.8	11.9	274	38.3	38.3	0.0	265	67.2	75.1	7.9	286	73.4	70.7	− 2.7
***Additional active treatment***																												
Surgery to surgery and EBRT (± ADT)	246	23.2	28.9	5.7	246	6.1	11.0	4.9	246	27.6	32.9	5.3	245	36.3	35.9	− 0.4	244	40.6	39.3	− 1.3	243	60.9	61.3	0.4	245	82.3	79.4	− 2.9
EBRT to EBRT and ADT	545	31.4	34.9	3.5	546	11.7	13.2	1.5	541	31.4	33.5	2.1	542	39.5	40.6	1.1	543	28.4	31.3	2.9	534	58.6	62.0	3.4	542	79.5	79.4	− 0.1
EBRT and ADT to surgery and EBRT (± ADT)	64	35.9	45.3	9.4	61	14.8	14.8	0.0	64	35.9	57.8	21.9	64	50.0	59.4	9.4	62	38.7	48.4	9.7	61	63.9	75.4	11.5	65	75.7	74.2	− 1.5
EBRT and ADT to systemic therapy and EBRT	116	36.2	49.1	12.9	117	12.8	21.4	8.6	117	41.0	55.6	14.6	116	55.2	64.7	9.5	117	42.7	50.4	7.7	115	73.9	79.1	5.2	115	75.5	71.6	− 3.9
ADT to systemic therapy and ADT	137	40.2	52.6	12.4	137	11.0	20.4	9.4	137	43.1	62.0	18.9	137	46.0	53.3	7.3	135	33.3	37.0	3.7	135	72.6	75.6	3.0	136	75.5	72.3	− 3.2
ADT to EBRT and ADT	84	36.9	50.0	13.1	84	10.7	13.1	2.4	83	41.0	49.4	8.4	83	47.0	50.6	3.6	85	44.7	27.1	− 17.6	81	70.4	64.2	− 6.2	82	76.5	73.9	− 2.6

Due to the large number of men included in the study, statistical significance can be achieved with only small differences in outcomes, and these may not be clinically relevant. Results should be considered alongside previously estimated clinically meaningful differences. For EQ-5D: self-assessed health (7 points)

*EBRT* external beam radiotherapy, *ADT* androgen deprivation therapy

^*^Includes surgery alone and surgery with EBRT and ADT

^**^Includes EBRT, EBRT and ADT and brachytherapy treatments

^***^Includes ADT alone, EBRT and ADT, ADT and systemic and surgery and EBRT and ADT treatments

### Respondents on monitoring at first survey who then received active treatment

#### Urinary and bowel function

In the group who reported surgery as their first active treatment at follow-up (alone or combined with EBRT and ADT), there was a CMD in the reporting of urinary incontinence: mean scores decreased by 11.9 points, indicating poorer function (Table 2). Worse urinary irritation and bowel function were reported by men whose first active treatment included EBRT (alone or combined with ADT) or brachytherapy. CMDs in mean urinary irritation and bowel function scores were observed at follow-up (− 6.6 points and − 8.3 points, respectively).

#### Vitality and hormone function

Declines in hormone function were reported by men who had moved to an active treatment at follow-up. These declines were largest (around 10 points) for men who had treatment involving EBRT, brachytherapy or ADT (either alone, combined or with systemic treatment) (Table 2).

#### Sexual function

A marked decline in sexual function was reported by all groups who reported their first active treatment at follow-up (Table 2). Mean sexual function scores in men who reported moving to EBRT (alone or combined with ADT) or brachytherapy were 17.9 points lower at follow-up (decreasing from 46.5 to 28.6). In men whose first active treatment was surgery, scores were on average 24.9 points lower at follow-up (decreasing from 50.5 to 25.6).

#### Generic HRQL

Patients who had ADT, EBRT or brachytherapy as their first active treatment reported more problems with all EQ-5D dimensions at follow-up. For example, 75.1% of ADT patients reported ≥ 1 problem at follow-up (a 7.9% increase) and 67.7% of EBRT or brachytherapy patients reported ≥ 1 problem at follow-up (a 12.3% increase). These issues do not appear to impact on SAH, with no clinically meaningful changes in scores observed (Table 4).

### Respondents who reported additional active treatment at follow-up

#### Urinary and bowel function

CMDs (declines) in urinary incontinence scores were observed for men who reported EBRT and ADT in the first survey and either surgical or systemic treatments at follow-up (− 6.0 points and − 6.7 points, respectively), although these groups were relatively small (Table 2). Men who reported the addition of EBRT reported worse bowel function at follow-up, with a CMDs for men treated with EBRT following initial ADT (− 5.8 points) and men treated with combined EBRT and ADT following surgery (− 6.7 points) (Table 2).

#### Vitality and Hormone function

Men who reported additional combined EBRT and ADT at follow-up, having previously reported only surgical treatment, reported a clinically meaningful 5 point decline in hormone function (Table 2). Men who reported a change in treatment from combined EBRT and ADT (likely ceasing treatment) to surgical treatment report an improvement in hormone function (+ 4 points, from 67.6 to 71.6).

#### Sexual function

Mean scores for sexual function declined between surveys in all treatment groups, but the differences observed were not clinically meaningful (Table 2).

#### Generic HRQL

Overall, men who received additional treatment between surveys reported more HRQL problems at follow-up (across all EQ-5D dimensions and SAH) (Table 4). The lowest SAH ratings and largest reductions in SAH were observed in men moving to systemic treatments (− 3.2 point change for men reporting ADT and systemic therapy at follow-up and − 3.9 point change for men reporting EBRT and systemic therapy at follow-up).

## Discussion

We report on a follow-up of the largest PCa patient-reported outcome study in the world to date, evaluating the on-going HRQL of the LAPCD cohort. Evidence from this study suggests stability of HRQL and most specific morbidities by 18–42 months for men who report no further active treatment in the subsequent 12 months, including those with advanced disease. However, this includes men who reported poor function in the original survey and continued to report poor function. At follow-up, 10% of the cohort reported receiving additional treatment or their first active treatment, which for many will be as a result of disease recurrence or progression.

The largest improvements in function were observed for hormonal issues, such as weight change, hot flushes, fatigue and depression. These are well-known side effects of ADT and were shown to be a major problem for men in the first LAPCD survey [14]. A clinically meaningful improvement in hormone function was observed in men who reported no further treatment following combined EBRT and ADT. It is documented that some men find their side effects improve or become more manageable the longer they are on ADT, while others find that side effects improve once they have stopped therapy and testosterone levels rise, although this can take several months or years [26]. It is therefore plausible that improvements were reported by this cohort because they either stopped or became accustomed to the impact of ADT. Unfortunately, we do not have data relating to the length of time that men were on ADT.

Although PCa-specific outcomes were stable in the year following the first survey, this includes continued poor sexual function across all treatment and sociodemographic cohorts. When looking at men who had completed both surveys and who reported no additional treatment at follow-up, few reported an improvement in function 12 months later. The lack of access to interventions to aid sexual function has been highlighted through the LAPCD study [14].

Another group requiring continued support is men experiencing poor urinary function, which is common following surgery. Almost three-quarters of surgical patients who reported poor continence in the first survey did not improve over the next year. Longitudinal research has shown that by 48 months, post-diagnosis urinary incontinence scores were significantly worse in surgical patients compared to other patient cohorts [27]. A study which followed patients for 15 years found that while urinary incontinence was less prevalent than sexual dysfunction, it was a greater cause of bother [11]. Support can be offered in a variety of ways, and possibilities for improving continence exist, including bladder retraining, pelvic floor exercises and medical interventions [28]. Clinicians should be encouraged to ask about urinary function in follow-up clinics, and men should be informed of the risk of longer-term quality of life issues.

Our results additionally identify a requirement to continue to support men undergoing ADT treatment. Previous research based on the LAPCD cohort has shown that worse cancer-specific and generic HRQL is associated with psychological distress and poor mental wellbeing in men treated with ADT [29]. This further emphasises the wider impact of cancer-specific HRQL. To date, interventions with ADT patients focus on lifestyle changes to reduce side effects and risk of developing further comorbidities from treatment [30].

Despite continued issues with urinary, bowel and sexual issues, overall, there was little change in HRQL among any of the treatment cohorts. These findings lend more support to the idea of the ‘gap hypothesis’ or ‘response shift’ of HRQL, the theory that being diagnosed with a life-threatening illness may result in patients re-evaluating what is important to them and re-calibrating expectations of what life with cancer will be like [31, 32].

Our results indicated that, of the 588 men who reported being on monitoring at initial survey and active treatment at follow-up, 39.8% reported subsequent surgery (alone) and 29.3% reported moving to ADT (alone). When compared to figures reported by PROTECT, which reported on men diagnosed with early stage disease only, our results are consistent for surgery, where half of the PROTECT cohort who started on monitoring moved to surgical treatment [10]. However, our results showed that only 5.6% of men were treated with radiotherapy alone after monitoring, which differ substantially from those reported by PROTECT, where a third of the cohort moved to radiotherapy after monitoring. These differences are not unexpected due to the LAPCD study including patients with both early stage and advanced disease, with many men receiving combined EBRT and ADT.

Treatment information was self-reported at both the initial and follow-up surveys. As such, there were some difficulties in trying to categorise responses into either ‘no further treatment’ or ‘additional treatment’. For example, many men reported an active treatment in the original survey but then reported active surveillance or watchful waiting at follow-up, which we interpreted as clinical monitoring and therefore no further active treatment. There were also instances where we believe that men reported only their current treatment in the follow-up survey rather than all received treatments. Due to these difficulties, some respondents had to be excluded from analyses.

There were some limitations in data interpretation. The first was that, due to privacy restrictions, we were unable to access date of diagnosis. This meant that outcomes could not be stratified by time since diagnosis, as information was not available as to how far post-diagnosis men were. The men included in this study are therefore a heterogenous group of medium- to long-term survivors. The second was that we did not have information about disease progression in the time since the first survey, as such data is not currently captured accurately by cancer registries. Finally, we were not definitively able to identify which men had finished treatment, which men were still receiving treatment and when they had last been treated. These factors will have some impact on HRQL outcomes but could not be investigated fully in this current study. Future research would benefit from recording this information as it may assist in providing greater understanding why some men experience worse or continued poor HRQL.

## Conclusions

Overall, patient-reported outcomes in men with PCa remained generally stable, which is reassuring for those with good function and HRQL but re-enforces the need for early intervention and support for those who experience poor outcomes, as these seem to persist for the majority. There remains a specific need to provide on-going support to men who have undergone ADT or surgical treatments as a high proportion of them report persistent problems. Poorer HRQL and specific functional problems were reported by men who received additional treatment and men who received their first active treatment between the two surveys: essentially, these patients start back at the beginning on their HRQL journey. These results further highlight that men living with and beyond PCa require patient-centred services to address treatment side effects, with the goal of enhancing their HRQL.

## Supplementary Information

Below is the link to the electronic supplementary material.

Supplementary file1 (DOCX 20 KB)

## Data Availability

The individual patient-level data used to generate results are not freely available but may be applied for through the Public Health England Office for Data Release.

## Code availability

Stata code is available from the authors upon request.
