# Supplementary material for: Stability of health-related quality of life and morbidity burden from 18 months after diagnosis of prostate cancer: results of a UK-wide population-based outcome cohort
Source: Support Care Cancer. 2021 Dec 13;30(4):3151–64. doi: 10.1007/s00520-021-06650-7 (PMC8857149; doi:10.1007/s00520-021-06650-7)
Supplement: Supplementary file 1 — Supplementary file1 (DOCX 20 KB) [file 520_2021_6650_MOESM1_ESM.docx]

**Supplementary table 1. Unverified patient-reported treatment changes (n=296)**

| **Treatment reported in survey 1** | **Treatment reported in survey 2** |
| --- | --- |
| Systemic & ADT | EBRT & ADT |
| Systemic & ADT | Surgery & EBRT/ADT |
| Systemic & ADT | EBRT |
| Systemic & ADT | Surgery |
| ADT | Surgery |
| ADT | EBRT |
| ADT | Brachytherapy |
| Brachytherapy | EBRT |
| Brachytherapy | Surgery |
| Brachytherapy | EBRT & ADT |
| Brachytherapy | ADT alone |
| Brachytherapy | Surgery & EBRT/ADT |
| Brachytherapy | Systemic & ADT |
| EBRT & ADT | Brachytherapy |
| EBRT & ADT | ADT & systemic |
| EBRT & ADT | Surgery |
| Systemic & EBRT | Brachytherapy |
| Systemic & EBRT | Surgery & EBRT/ADT |
| EBRT | Brachytherapy |
| EBRT | ADT |
| EBRT | Surgery |
| EBRT | Systemic & ADT |
| Surgery & EBRT/ADT | Systemic & ADT |
| Surgery & EBRT/ADT | Systemic & EBRT |
| Surgery & EBRT/ADT | Brachytherapy |
| Surgery | Brachytherapy |
| Surgery | ADT |
| Surgery | EBRT |
| Surgery | EBRT & ADT |
| Surgery | Systemic & ADT |

EBRT: External beam radiotherapy; ADT: Androgen deprivation therapy

**Supplementary table 2. Categorisation of patient-reported treatment changes**

| **Treatment** | **Treatment reported in survey 1** | **Treatment reported in survey 2** | **Total** |
| --- | --- | --- | --- |
| **No additional treatment (n=19,470)** | ***Monitoring*** | | 3,039 |
|  | AS/WW | AS/WW |  |
|  | ***Surgery*** | | 5,311 |
|  | Surgery | Surgery |  |
|  | Surgery | AS/WW |  |
|  | ***EBRT*** | | 1,030 |
|  | EBRT | EBRT |  |
|  | EBRT | AS/WW |  |
|  | ***Brachytherapy*** | | 766 |
|  | Brachytherapy | Brachytherapy |  |
|  | Brachytherapy | AS/WW |  |
|  | Brachytherapy | WW |  |
|  | ***ADT*** | | 1,618 |
|  | ADT | ADT |  |
|  | ADT | AS/WW |  |
|  | ***EBRT & ADT*** | | 5,483 |
|  | EBRT & ADT | EBRT & ADT |  |
|  | EBRT & ADT | EBRT |  |
|  | EBRT & ADT | ADT |  |
|  | EBRT & ADT | AS/WW |  |
|  | ***Surg & EBRT/ADT*** | | 1,651 |
|  | Surgery & EBRT/ADT | Surgery & EBRT/ADT |  |
|  | Surgery & EBRT/ADT | EBRT & ADT |  |
|  | Surgery & EBRT/ADT | AS/WW |  |
|  | Surgery & EBRT/ADT | ADT |  |
|  | Surgery & EBRT/ADT | Surgery |  |
|  | Surgery & EBRT/ADT | EBRT |  |
|  | ***ADT & systemic*** | | 303 |
|  | ADT & systemic | ADT & systemic |  |
|  | ADT & systemic | ADT |  |
|  | ADT & Systemic | AS/WW |  |
|  | ***EBRT & systemic*** | | 269 |
|  | EBRT & systemic | EBRT & systemic |  |
|  | EBRT & systemic | EBRT |  |
|  | EBRT & systemic | ADT & systemic |  |
|  | EBRT & systemic | EBRT & ADT |  |
|  | EBRT & Systemic | AS/WW |  |
| **Verified treatment changes (n=1,934)** | ***AS/WW to first active treatment*** | | 588 |
|  | AS/WW | Surgery |  |
|  | AS/WW | ADT |  |
|  | AS/WW | EBRT & ADT |  |
|  | AS/WW | EBRT |  |
|  | AS/WW | Systemic & ADT |  |
|  | AS/WW | Brachytherapy |  |
|  | AS/WW | Surgery & EBRT/ADT |  |
|  | AS/WW | Systemic & EBRT |  |
|  | ***Additional active treatment*** | | 1,346 |
|  | ADT | Systemic & ADT |  |
|  | ADT | EBRT & ADT |  |
|  | ADT | Systemic & EBRT |  |
|  | ADT | Surgery & EBRT/ADT |  |
|  | ADT & systemic | Systemic & EBRT |  |
|  | EBRT | EBRT & ADT |  |
|  | EBRT | Systemic & EBRT |  |
|  | EBRT & ADT | Systemic & EBRT |  |
|  | EBRT & ADT | Surgery & EBRT/ADT |  |
|  | EBRT | Surgery & EBRT/ADT |  |
|  | Surgery | Surgery & EBRT/ADT |  |

AS: Active surveillance; WW: Watchful waiting; EBRT: External beam radiotherapy; ADT: Androgen deprivation therapy
